# Nonionic Amphiphilic Copolymers of Poly(poly(ethylene Glycol) Methacrylate) Brushes with Methyl Methacrylate Prepared by Atom Transfer Radical Polymerization as Dry Solid Polymer Electrolytes for Next Generation Li-ion Battery Applications

**DOI:** 10.1021/acsaem.4c02519

**Published:** 2024-12-09

**Authors:** Ákos Szabó, Denis Ershov, Ágnes Ábrahám, Éva Kiss, Györgyi Szarka, Ilona Felhősi, Benjámin Gyarmati, Attila Domján, Béla Iván, Robert Kun

**Affiliations:** a Polymer Chemistry and Physics Research Group, Institute of Materials and Environmental Chemistry, HUN-REN Research Centre for Natural Sciences, Magyar tudósok krt. 2., Budapest H-1117, Hungary; b Department of Chemical and Environmental Process Engineering, Faculty of Chemical Technology and Biotechnology, Budapest University of Technology and Economics, Műegyetem rkp. 3, Budapest H-1111, Hungary; c MTA-TTK Lendület “Momentum” Peptide-Based Vaccines Research Group, Institute of Materials and Environmental Chemistry, HUN-REN Research Centre for Natural Sciences, Budapest H-1117, Magyar tudósok krt. 2.; d Laboratory of Interfaces and Nanostructures, Institute of Chemistry, Eötvös Loránd University, 112, PO Box 32, Budapest H-1518, Hungary; e Functional Interfaces Research Group, Institute of Materials and Environmental Chemistry, HUN-REN Research Centre for Natural Sciences, Magyar tudósok krt. 2., Budapest H-1117, Hungary; f Soft Matters Group, Department of Physical Chemistry and Materials Science, Faculty of Chemical Technology and Biotechnology, Budapest University of Technology and Economics, Műegyetem rkp. 3, Budapest H-1111, Hungary; h Centre for Structural Science, HUN-REN Research Centre for Natural Sciences, Magyar tudósok krt. 2, Budapest 1117, Hungary; i Solid-State Energy Storage Research Group, Institute of Materials and Environmental Chemistry, HUN-REN Research Centre for Natural Sciences, Magyar tudósok krt. 2., Budapest H-1117, Hungary

**Keywords:** DSPE, Li-ion battery, ATRP, MAS NMR, copolymer electrolyte, comb-like poly(poly(ethylene glycol) methacrylate) (PPEGMA) copolymer with MMA

## Abstract

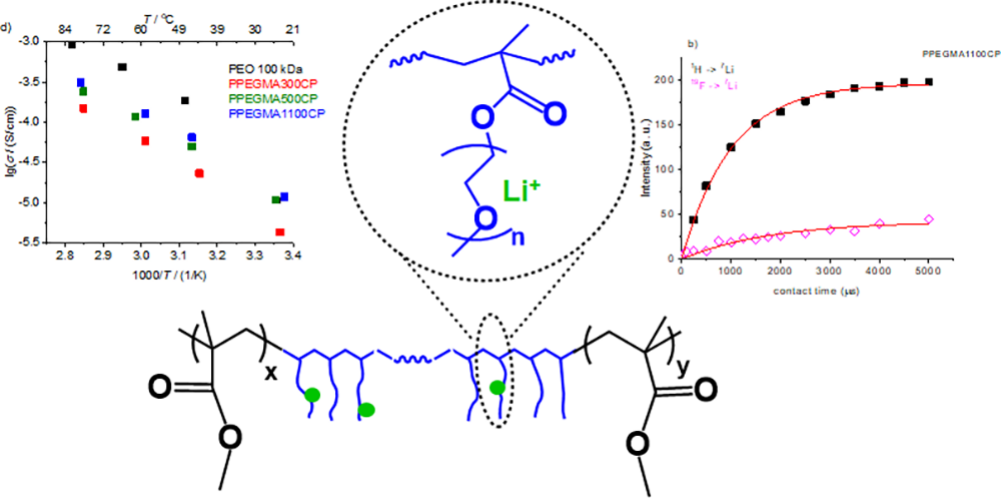

Amphiphilic copolymers of comb-like poly(poly(ethylene glycol) methacrylate) (PPEGMA) with methyl methacrylate (MMA) synthesized by one-pot atom transfer radical polymerization were mixed with lithium bis (trifluoromethanesulfonyl) imide salt to formulate dry solid polymer electrolytes (DSPE) for semisolid-state Li-ion battery applications. The PEO-type side chain length (EO monomer’s number) in the PEGMA macromonomer units was varied, and its influence on the mechanical and electrochemical characteristics was investigated. It was found that the copolymers, due to the presence of PMMA segments, possess viscoelastic behavior and less change in mechanical properties than a PEO homopolymer with 100 kDa molecular weight in the investigated temperature range. In contrast to the PEO homopolymer, it was found that no crystallization of the copolymers occurs in the presence of the Li-salt. Solid-state NMR and cross-polarization NMR studies revealed that no crystallization (i.e., ion-pair formation) of the Li-salt occurs in the case of the copolymer samples at ambient temperatures; thereby, no phase separation takes place, in contrast to the reference PEO homopolymer sample, which resulted in fairly good ionic conductivity of the copolymers at lower temperatures. The temperature-dependent Li-ion conductivity analyses showed that the conductivity of the copolymers falls in the 10^–6^–10^–3^ S/cm range, which is typical for polyether-type DSPEs, but the much lower mass fraction of EO monomers in the copolymers provides the same ionic conductivity values than that of the PEO homopolymer. From a large-scale practical point of view, this clearly indicates reduced Li-salt usage if such copolymer matrices are used instead of PEO homopolymer. Moreover, linear sweep voltammetry (LSV) polarization measurements showed that the PPEGMA-MMA copolymer electrolytes can exhibit a 200–300 mV broader electrochemical stability window than the PEO homopolymer, which is crucial in designing high energy density semisolid-state Li-ion batteries.

## 1 Introduction

Recently, Li-ion batteries have been the foremost used electrochemical energy storage devices in many applications. However, their safety and electrochemical performance must be improved to fulfill the growing consumer and market needs. Next-generation Li-ion batteries may possess better energy and power density performance combined with excellent safety attributes. Ceramic, glassy, and polymer-type Li-ion electrolyte-based solid-state batteries are regarded as the next step in the electrochemical energy storage science and technology.^1^ One of the most promising and broadly investigated polymer electrolyte candidates is poly(ethylene oxide) (PEO).^2,3^ However, to fulfill the requirements of the solid polymer electrolytes (SPEs) used in such next-generation polymer-type lithium–metal batteries (LMB), the drawbacks related to the linear PEO should be overcome. This includes the crystalline phase formation at ambient temperatures, which decreases considerably the specific ionic conductivity due to the sluggish segmental motion of the polymer chains since segment dynamics is directly related to the Li-ion movement in the bulk polymer. Another challenging issue is the significant change in the mechanical properties below and above the PEO’s melting point (> ∼ 54 °C), which results in a liquid-like behavior of the polymer electrolyte at elevated temperatures.^4^ While the suppression of the semicrystalline nature of the PEO matrix can be achieved by nanocomposite formulation or the addition of organic plasticizers,^5−8^ these strategies only slightly influence or even worsen the mechanical properties of the polymer electrolyte. Moreover, migration of the plasticizer and phase separation phenomena should also be considered. Another way can be the variation of the macromolecular structure, e.g. synthesizing highly branched PEO derivatives.^9−12^ Poly(poly(or oligo) (ethylene glycol) methacrylate)s (PPEGMAs) are comb-like (or brush or flask-brush-type) macromolecules with a polymethacrylate backbone and a large number of short poly(ethylene glycol) side chains, i.e., a pendant PEG chain on every monomeric unit.^13^ Due to this special structure, these polymers show unique physical-chemical properties, such as thermoresponsive and antifouling behavior, as well. Owing to their comb-like structure, the crystallinity of the PEG side chains is suppressed in PPEGMAs related to the linear PEG polymers.^14,15^ Due to the methacrylate functional group of the monomers, they can also be synthesized by quasiliving polymerization techniques, e.g., atom transfer radical polymerization (ATRP), which makes the formation of tailored chain structures possible.^16−19^ Moreover, the PEGMA macromonomers can also be copolymerized with many other monomers opening another way to fine-tune the properties of their polymer derivatives.^20^ The application of numerous homo- and copolymers of PEGMAs as possible Li-ion battery electrolytes has been proposed previously.^3,12,18,21−31^ For example, Wang et al. synthesized PPEGMAs with different side chain lengths by free radical polymerization and studied their ionic conductivity at a fixed lithium bis(trifluoromethanesulfonyl)imide (LiTFSI) content.^32^ They experienced higher specific conductivities in the case of longer side chains.

Investigating the mechanism of Li-ion conduction in PPEGMA-s, Patel and co-workers found on the basis of vibrational spectroscopy data that LiTFSI in the case of EO:Li^+^ = 20 molar ratio is fully dissociated in a pure PPEGMA matrix, and Li ions are coordinated by etheric O atoms of the side chains. They concluded that these salt-polymer systems show higher specific conductivity in the case of longer polyether side chain length and showed by simulations that the side chain etheric O atoms far from the backbone have higher Li^+^-complexing ability and faster dynamics.^33^ Gilbert et al. synthesized PS–PPEGMA AB block copolymer and proved by XPS measurements that the distribution of the Li and F atoms is correlated, and LiCF_3_SO_3_ salt is uniformly distributed in the PPEGMA domains of the formed polymer nanostructure,^27^ in contrast to PS–PEO block copolymers with linear PEO blocks,^34^ indicating the less confined dynamics of the PEO segments in a side chain than in the backbone coupled to a hard segment. Bennington et al. synthesized the block and random copolymers of PEGMA and glycerol carbonate methacrylate and found that mainly the PEGMA side chains coordinate the lithium ions in these copolymers.^35,36^

To improve the mechanical properties of the polymer electrolytes, which is essential in a functional semisolid-state Li-ion battery, hard segments were coupled to the PPEGMA chains, such as polystyrene^23,37^ or poly(methyl methacrylate) (PMMA). The latter has a similar structure as the PPEGMA’s backbone and the same living polymerization techniques can be used for its synthesis,^18,19,24^ which proves the relatively easy formation of their block copolymers. This process was successfully applied to synthesize poly(methyl methacrylate)–poly(poly(ethylene glycol) methacrylate)-polyisobutylene ABCBA pentablock terpolymers, which form physical networks at room temperature.^17^ Mayes and co-workers synthesized PMMA-*b*-PPEGMA diblock copolymers by anionic polymerization,^29−31^ and found that the addition of Li-trifluoromethylsulfonate enhances the microphase separation.^29^ Moreover, the combination of PMMA blocks with PPEGMA blocks results in a polymer matrix with mixed hydrophilicity/hydrophobicity, which is expected to be advantageous in the aspect of the desired intimate contact with both hydrophilic and hydrophobic fillers. Bergfelt et al. prepared PMMA–PPEGMA-PMMA ABA triblock copolymers and studied their Li-ion conductivity.^19^ They proved by SANS measurements that a mixed phase is formed in these materials.

The objective of the present work is the synthesis and investigation of copolymer electrolytes of poly(poly(ethylene glycol) methacrylate) (PPEGMA) brushes modified with poly(methyl methacrylate) (PMMA) segments by a one-pot ATRP process using PEGMA macromonomers with different side chain length, i.e., with molar masses of 300, 500, and 1100 g/mol. The surface characteristics of these copolymers were also studied by wetting experiments. Additionally, the behavior of such copolymers and linear PEO, both mixed with lithium bis(trifluoromethanesulfonyl)imide (LiTFSI) salt to obtain Li-ion conducting electrolytes, were compared by rheometric and solid-state NMR measurements to better understand the effect of the polymer structure on the Li-ion conduction mechanism. These investigations serve as the expansion of the chemical toolkit with which dry solid-state polymer electrolytes (DSPE) can be prepared with tunable mechanical and mixed polarity character.

## 2 Experimental Section

### 2.1 Materials

Poly(ethylene glycol) (PEG400), poly(ethylene glycol) methacrylates with *M* = 300 (PEGMA300), 500 (PEGMA500), and 1100 (PEGMA1100) g/mol, lithium bis(trifluoromethanesulfonyl)imide (LiTFSI) (99.95%), ascorbic acid, 2-bromoisobutyryl bromide, ethyl 2-bromoisobutyrate, 1,1,4,7,10,10-hexamethyl-triethylenetetraamine (HMTETA), CuCl, Li metal, acetonitrile, and poly(ethylene oxide) (*M* = 100 000 g/mol) were purchased from Sigma-Aldrich. The inhibitors were removed from PEGMA and methyl methacrylate monomers by passing them through a column filled with basic Al_2_O_3_. CuCl was stirred with acetic acid overnight, filtered, and washed with abs. ethanol and diethyl ether.

### 2.2 Methods

#### 2.2.1 Sample Synthesis

The preparation of the α,ω-bis(2-bromoisobutyrate)-telechelic poly(ethylene glycol) initiator (Br-PEG-Br) is described in the Supporting Information (see Figure S1 for its ^1^H NMR spectrum). For the synthesis of PPEGMA500CP copolymer (the other copolymers were synthesized by an analogous way), 105.3 mg (0.15 mmol) of the Br-PEG-Br initiator (*M*(initiator) = 698 g/mol), 107.7 mg of l-ascorbic acid, 32.4 mg (0.33 mmol) of CuCl, 3.0 mL of toluene, and 1.40 mL (3.0 mmol) of inhibitor-free PEGMA500 was added to a glass vial closed with a rubber septum. Then, Ar gas was bubbled through the reaction mixture for 10 min. Then, 0.08 mL (0.29 mmol) of 1,1,4,7,10,10-hexamethyltriethylene tetramine was added. The polymerization was started by heating the vial to 80 °C. After 5 h of polymerization at 80 °C, 0.45 mL of sample was withdrawn, and 1.44 mL (13 mmol) of methyl methacrylate was added followed by 1 min of Ar bubbling. The reaction mixture was kept at 80 °C for another 18 h. Subsequently, the reaction mixture was cooled to room temperature, diluted with toluene, passed through a column filled with neutral Al_2_O_3_ followed by the evaporation of the solvent and unreacted methyl methacrylate, and dried in vacuum at room temperature and at 60 °C. The same process was applied for the withdrawn sample as well.

#### 2.2.2 Electrochemical Cell Assembly

For the electrochemical characterization of the dry polymer electrolytes, the cells were assembled under an inert atmosphere (nitrogen or, in the case of Li metal electrodes, in argon). The dry polymer electrolyte films were tape cast by dropping their LiTFSI-containing solution in acetonitrile into the 6 mm inner diameter hole of a 10 mm outer diameter Teflon spacer put on a 10 mm diameter disk of the electrode metal stainless steel (SS) foil used as “blocking electrode”. Due to the reaction between acetonitrile and lithium, the dry polymer-salt mixture was transferred by a spatula into the spacer hole in the case of lithium metal foil as a “reversible electrode”. After evaporation of the solvent, the electrode/DSPE stacks were put into vacuum for 30 min followed by closing with the other electrode disk (either SS or Li) with the same size forming the symmetrical cell (i.e., SS/DSPE/SS or Li/DSPE/Li) into a 10 mm inner diameter glass tube between two metal joints equipped with a springbased compressing mechanism.

#### 2.2.3 Characterizations

For the thermal characterizations of the polymer matrices and the Li-ion conductive polymer electrolyte samples, differential scanning calorimetry (DSC) was used. DSC curves were recorded on a Mettler DSC30 measuring apparatus equipped with a Mettler TA15 controller. The analyses were carried out in the 173–473 K temperature range using a 10 K/min heating rate. According to the measurement protocol, a few milligrams of the polymeric sample was placed into an Al crucible and sealed with an Al lid in an inert glovebox environment. The closed crucibles along with the polymer specimen were heated and cooled down in the applied temperature range followed by the repeated heating of the specimen without removing this from the measurement apparatus. The obtained enthalpograms of the second heating cycle were evaluated afterward. The crystalline fraction of PEO in the PEO:LiTFSI salt mixture was estimated by the following equation:
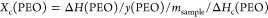
1where *X*_c_(PEO) is the crystalline fraction of PEO, Δ*H*(PEO) is the integral of the melting peak on the calorimetric curve, *y*(PEO) is the weight ratio of PEO in the PEO-salt mixture, *m*_sample_ is the mass of the DSC sample, and Δ*H*_c_(PEO) is the specific heat of the crystallization of PEO (−197 J/g^38^).

The gel permeation chromatography (GPC) measurements were carried out with a setup composed of a Waters 515 HPLC pump, a column system of two Waters Styragel HR columns (HR1 and HR4), Jetstream thermostat, and an Agilent Infinity 1260 differential refractometer detector. Tetrahydrofuran was used as the eluent with a flow rate of 0.3 mL/min, and the column temperature was 35 °C.

Solid-state magic angle spinning nuclear magnetic resonance (MAS NMR) spectra of the samples were recorded by a Varian System spectrometer operating at 399.9 MHz for the ^1^H frequency (155.4 MHz for ^7^Li, 376.2 MHz for ^19^F and 100.6 MHz for ^13^C) with a Chemagnetics 4.0 mm narrow-bore double resonance T3 probe. Rotors were filled in a glovebox, sealed against air and moisture tightly, and placed immediately into the spectrometer. The 90° pulse lengths were 5.4 μs for ^7^Li and ^13^C and 4.0 μs for the proton (^1^H, ^19^F) channel. Temperature was calibrated previously with Pb(NO_3_)_2_ at different spinning rates. Solution-state routine NMR spectra of the synthesized polymers, dissolved in CDCl_3_, were recorded with a 500 MHz Varian system spectrometer.

Electrochemical impedance (EIS) measurements were performed on a Zahner IM6e frequency analyzer. The applied frequency range was 1 to 10^5^ Hz, and the amplitude was 10 mV. Nine points per frequency decade were recorded with 25 (above 66 Hz) or 10 (below 66 Hz) scans. The fitting of the data was carried out by the program ZView. For the electrochemical stability measurements, a Biologic VMP-300 potentiostat was applied between 0 and 6 V voltage using a symmetrical cell assembly equipped with stainless steel blocking electrodes.

Li^+^ transference number was measured on the basis of the method of Evans et al.^39^ using a symmetrical Li/DSPE/Li cell at 70 °C. The bulk resistance before and after polarization was determined by EIS measurements. For the polarization, 10 mV voltage was applied to the cell, while the current was monitored in the function of time during 20 h.

The storage and loss moduli were determined by oscillation rheometry measurements performed on an Anton Paar Physica MCR 301 rheometer with a cone–plate geometry probe (diameter: 25 mm; cone angle: 1°; sample gap: 0.054 mm) applying 1% strain in the 0.5–500 1/s angular frequency range.

Contact angle measurements were performed on copolymer films cast on stainless steel foils.^42^ The copolymers were dissolved in either acetonitrile or an acetonitrile-dichloromethane mixture to obtain a homogeneous solution with a concentration of 20 g/dm^3^. From these solutions, 300 μL was spread onto the stainless-steel surface, which had been freshly rinsed (or cleaned) with acetone. After drying, the polymer samples were kept in a vacuum chamber for 24 h to allow the complete evaporation of the remaining solvent traces. The time-dependent contact angles of a 5 μL liquid droplet were measured using a goniometric method (OCA15+, Dataphysics, Germany) in a closed, solvent vapor saturated chamber. For every sample, 16 to 20 parallel measurements were performed. The relationship between the contact angle and the liquid surface tension (σ_L_), the liquid/solid interfacial tension (σ_SL_) and the surface energy (σ_S_) is defined by the Young equation:^40^
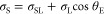
2

For surface energy estimation, the Wu model was used, which is suggested especially for polymer surfaces.^41^ Based on the Fowkes method, the interactions between a solid and a liquid phase are divided into dispersive and polar interactions. The σ_SL_ is interpreted as follows, including the harmonic mean of the dispersive and polar surface tension components:
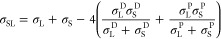
3where σ_L_^D^, σ_L_^P^, σ_S_^D^, and σ_S_^P^ stand for the dispersive and polar surface tension components of the liquid and surface energy components of the solid phases, respectively. To determine the solid surface energy, at least two liquids are necessary to measure the contact angles on the solid surface. In our case, we used water and ethylene glycol as polar, and *n*-dodecane as apolar solvents with known dispersed and polar parts of the surface tension.

## 3 Results and Discussion

**Scheme 1:**
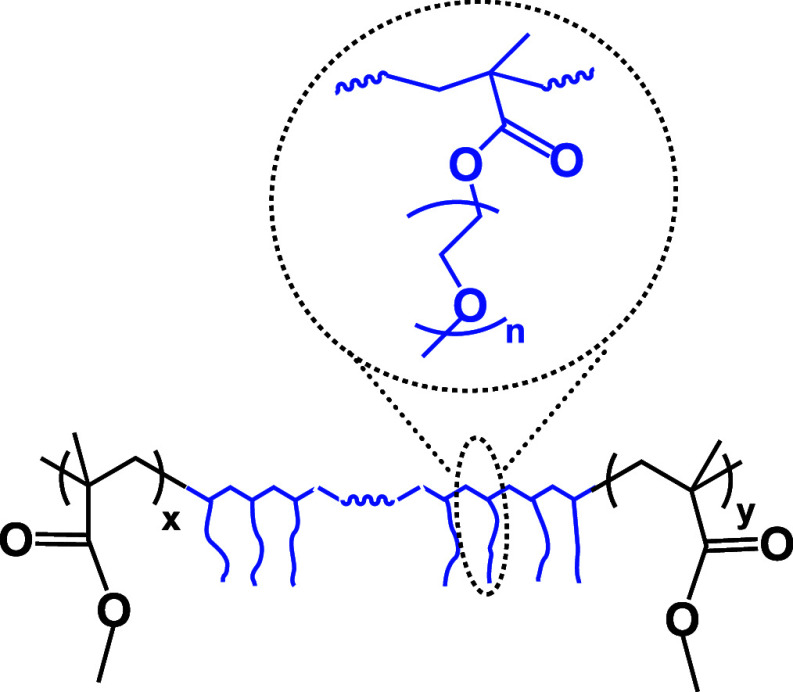
Structure of PMMA–PPEGMA-PMMA Triblock Copolymers

The applied one-pot ATRP process, that is direct addition of MMA in the reaction mixture and subsequent polymerization without isolating and purifying the formed PPEGMA brushes in the first step, is expected to lead to poly(methyl methacrylate)-*b*-poly(poly(ethylene glycol) methacrylate)-*b*-poly(methyl methacrylate) (PMMA–PPEGMA-PMMA) triblock copolymers as shown in Scheme 1. In these copolymers, the poly(ethylene glycol) side chains in the comb-like inner PPEGMA block provide the Li-ion conducting polyetheric matrix, and the PMMA segments are expected to be responsible for improved mechanical properties. Three different copolymers with different poly(ethylene glycol) side chain lengths, i.e., with number-average molecular weights of 300, 500, and 1100 g/mol, were synthesized. The resulting PMMA containing copolymers are denoted as PPEGMA300CP, PPEGMA500CP, and PPEGMA1100CP for samples containing PEGMA with molecular weights of 300, 500, and 1100 g/mol side chains, respectively. The GPC curves and ^1^H NMR spectra in Figures S2–S7 indicate the attachment of MMA units to the PPEGMA brushes. The GPC curves of the PPEGMA precursors and the product formed after the addition of MMA indicate partial formation of triblock copolymers and the presence of PPEGMA brushes presumably with few terminal MMA units, i.e. the applied process leads to the formation of the mixture of such macromolecular structures (Figures S2, S4, and S6). On the basis of the ^1^H NMR spectra (Figures S3, S5, and S7), copolymers with 10–20 wt % methyl methacrylate, i.e., with 80–90 wt % PEG contents are obtained (Table 1). Moreover, the formed copolymers, in contrast to the PPEGMA homopolymers, are not water-soluble due to the incorporated PMMA in the one-pot polymerization process.

**Table 1:** PEGMA Side Chain Lengths and the PEG Contents of the Synthesized Copolymers

sample	PEGMA molecular weight (g/mol)	average number of EO units in the side chains	weight ratio of PEG (%)
PPEGMA300CP	300	4.5	90
PPEGMA500CP	500	9	91
PPEGMA1100CP	1100	23	80

**Figure 1:**
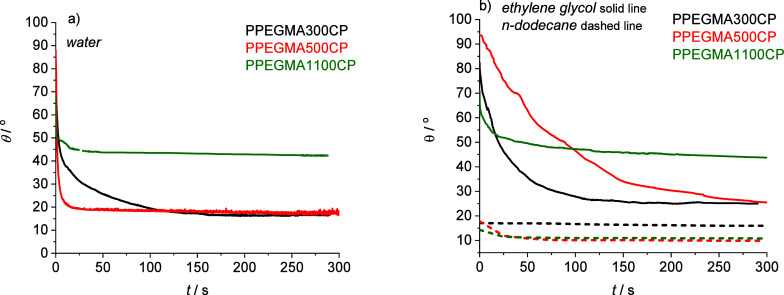
Contact angles of water (a), ethylene glycol and *n*-dodecane (b) of the copolymer films as a function of time.

In order to confirm the mixed polarity character of the as-synthesized copolymers, wetting property determination was carried out. Contact angle measurements were used to determine the wetting properties and the assumed mixed hydrophilic/hydrophobic surface characteristics of the copolymer samples. The wetting properties of the copolymer films cast on stainless steel foil were studied. In the initial instants of the measurements, the water contact angles of all of the copolymer films (PPEGMA300CP, PPEGMA500CP, PPEGMA1100CP) were found to range between 70 and 90°, indicating that they are rather hydrophobic. This suggests that the comb-like PPEGMA polymer chains are oriented in a way that the hydrophobic polymethacrylate backbone together with the PMMA segments forms the surface while the hydrophilic PEO side chains turn into the bulk phase. However, the investigation of the time-dependent change of the water contact angle (see Figure 1a) reveals that the PPEGMA300CP and PPEGMA500CP copolymer films exhibit significant surface characteristic changes within a few minutes, whereas the PPEGMA1100CP film shows a much smaller response. This observation indicates that the orientation of the polymer chains changes over time, and the hydrophilic PEG side chains turn toward the contacting water phase, resulting in a much more hydrophilic interface. In the PPEGMA1100CP film, the slower decrease in the contact angle can be attributed to the formation of a rigid crystalline phase of the PEG side chains, which couples the hydrophilic side chains to each other, hindering the surface rearrangement. This restriction in movement can result in a limited change in the orientation of the macromolecule, thereby affecting the surface characteristics.

**Figure 2:**
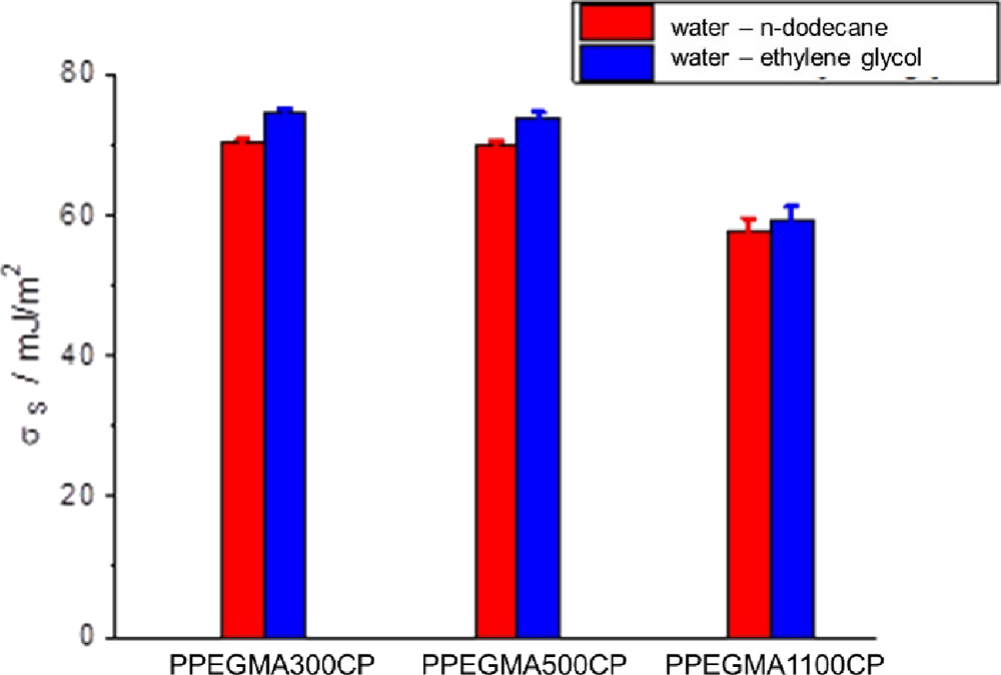
Solid surface energy values of the copolymer films after 5 min liquid contact determined by the Wu harmonic mean method from the contact angles in water, *n*-dodecane, and ethylene glycol.

The surface polarity was also studied by using different solvents with varying polarity, such as ethylene glycol and *n*-dodecane, to gain further insights into the specific interaction between the liquid and the polymer film as well as to determine the solid surface energy. The relative polarity values of these liquids are as follows: 1 for water, 0.79 for ethylene glycol, and 0.009 for *n*-dodecane.^43^ When ethylene glycol is used, which is also a thermodynamically good solvent for the PEO side chains, and a nonsolvent of the polymethacrylate backbone, the contact angle values initially range from 70 to 90°, but within 5 min, they decrease to the range of 25–50°. The least pronounced change is observed once again for the PPEGMA1100CP sample (Figure 1b). In contrast, when the nonpolar liquid, *n*-dodecane is used, which is nonsolvent for both the polymethacrylate backbone and the PEO side chains, the contact angles exhibit low values initially, and their changes over time are only moderate (Figure 1b). To obtain the solid surface energy values, which provide information about the hydrophilicity of the copolymer films, the Wu harmonic mean eq (eq 3) was employed, which involves the use of two different liquids.^41^ In comparison to the zero min surface energy (30–40 mJ/m^2^), the polarity of the polymer surface increases, reaching a value of 70 mJ/m^2^ for PPEGMA300CP and PPEGMA500CP after 5 min of contact with liquids. This indicates the formation of a highly hydrophilic surface.^40,41^ Additionally, a hydrophilic surface was observed in the case of PPEGMA1100CP characterized by a slightly lower surface energy (58 mJ/m^2^). The applied estimation method resulted in an increase in the polarity of the polymer surfaces upon contact with the liquids, particularly for PPEGMA300CP and PPEGMA500CP, which have shorter and potentially more mobile PEG side chains (Figure 2).

These findings suggest that amphiphilic poly(PEGMA)s with flexible and mobile chains can dynamically change their surface/interface properties relating to the contact phase above their glass transition temperature. In summary, the results of the wetting property measurements indicate that the investigated copolymer matrices have good wetting on both hydrophilic and hydrophobic surfaces; i.e., electrode additives/fillers with opposite surface polarities can be simultaneously applied in the same DSPE host.

In the case of poly(ethylene oxide) (PEO) type dry solid polymer electrolytes, the polymer (semi)crystal formation has a considerable effect on the Li-ion conductivity due to the segment coupling effect. Thereby, polymer segment dynamics is crucially influencing Li-ion motion in the polymer matrix.^44^ The formation of semicrystalline polymer phases decreases the polymer chain segment motion, which results in decreased Li-ion conductivity. Thermochemical properties of the obtained comb-like copolymers were examined by differential scanning calorimetry (DSC) measurements. It was expected that the comb-like structure suppresses the formation of the semicrystalline PEO domains in the synthesized copolymers.

**Figure 3:**
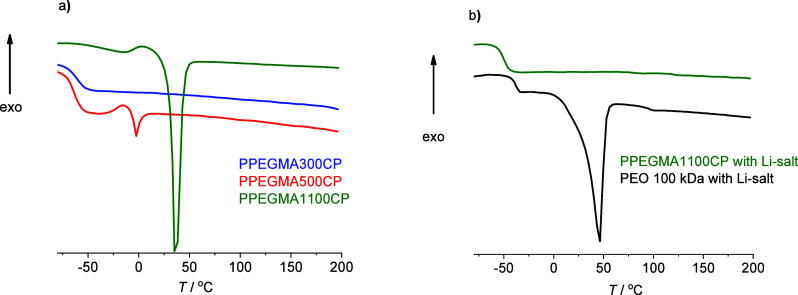
Differential scanning calorimetry (DSC) enthalpograms of the LiTFSI-free copolymers (a), and comparison of the enthalpograms for PPEGMA1100CP and 100 kDa PEO mixed with LiTFSI (EO:Li^+^ molar ratio = 16:1) (b).

The recorded DSC curves in Figure 3a show that the copolymers with PEO side chains having 9 or more EO units (PPEGMA500CP and PPEGMA1100CP) tend to partially crystallize. In contrast, the sample that contains only 4.5 EO units in the PEO side chain (PPEGMA300CP) can be considered as amorphous. However, the glass transition in the range characteristic of the PMMA (∼100 °C) cannot be observed for the investigated copolymers. This is in accordance with Bergfelt et al., who explained this by the presence of mixed phases,^19^ although the observed *T*_g_ values (−61 °C for PPEGMA300CP and −65 °C for PPEGMA500CP) are characteristic for the pure PPEGMA segments^14^ and are lower than that calculated by the Fox equation. The melting temperature of the formed semicrystalline polymer phases is much lower than the ambient temperature in the case of side chains with 9 EO units (PPEGMA500CP) (*T*_melting_ = −3 °C). However, in the case of side chains with 23 EO units (PPEGMA1100CP), the melting temperature (*T*_melting_ = 36 °C) is close to that of the PEO homopolymer. To demonstrate the effect of the Li-salt dosing on the thermal behavior of the polymer matrices, the enthalpogram of lithium bis(trifluoromethanesulfonyl)imide (LiTFSI) containing PPEGMA1100CP, compared to the LiTFSI enriched PEO 100 kDa, was recorded as shown in Figure 3b. The presence of Li-salt in the DSPE polymer matrix with semicrystalline character results in the suppression of the crystallinity.^21^ For the PEO 100 kDa, this is manifested by the decrease of the melting point from 64 to 46 °C by the addition of LiTFSI with an EO monomeric unit/Li^+^ = 16:1 molar ratio. The crystalline fraction of PEO in this polymer-salt mixture can be calculated by eq 1, yielding 37%. In sharp contrast, the DSC curve of the LiTFSI containing the PPEGMA1100CP electrolyte shows only the glass transition without any crystallization. This means that the formation of semicrystalline phases in the DSPE can be fully suppressed by using such comb-like polymeric structures.

**Figure 4:**
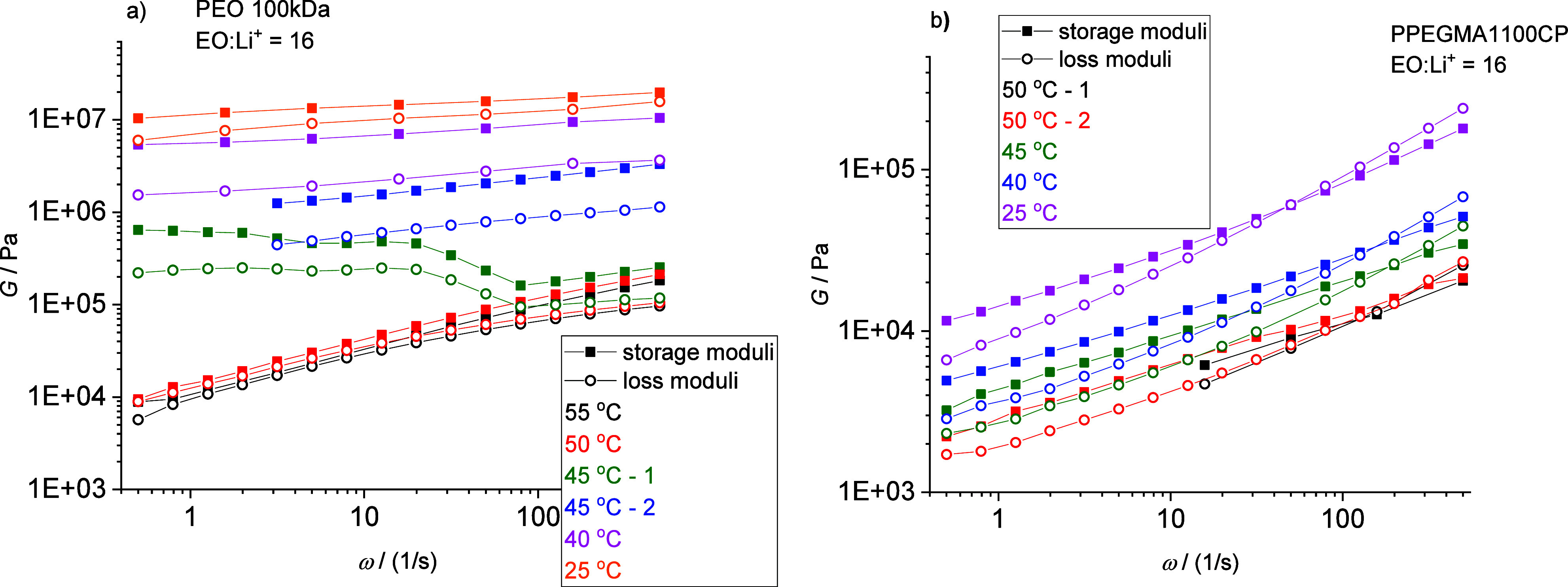
Storage and loss moduli obtained from oscillation rheometry measurements for the LiTFSI containing PEO (a) and PPEGMA1100CP (b) samples at different temperatures (EO: Li^+^ molar ratio = 16:1).

The temperature-dependent mechanical properties of the above-mentioned (i.e., Li-salt containing) polymers, investigated by oscillation rheometry, are also in accordance with the DSC results. In the case of the LiTFSI containing PEO 100 kDa, there is a 2 orders of magnitude increment in both the storage and loss moduli by cooling the mixture from 50 to 45 °C indicating the transition from the viscoelastic polymer melt to a solid-like semicrystalline polymer (Figure 4a). Above this transition, the polymer’s behavior can be estimated by the Rouse model, since *G*′ ≈ *G*′′ ≈ ω^0.4^ while below this transition the polymer behaves like a solid material with nearly frequency-independent moduli. In sharp contrast, the LiTFSI containing PPEGMA1100CP shows only a time–temperature superposition shift by changing the measurement temperature in this region, as displayed in Figure 4b. Assuming the power-like *G* ≈ ω^*k*^ relation, *k* = 0.3–0.5 for the LiTFSI containing PPEGMA1100CP.

**Figure 5:**
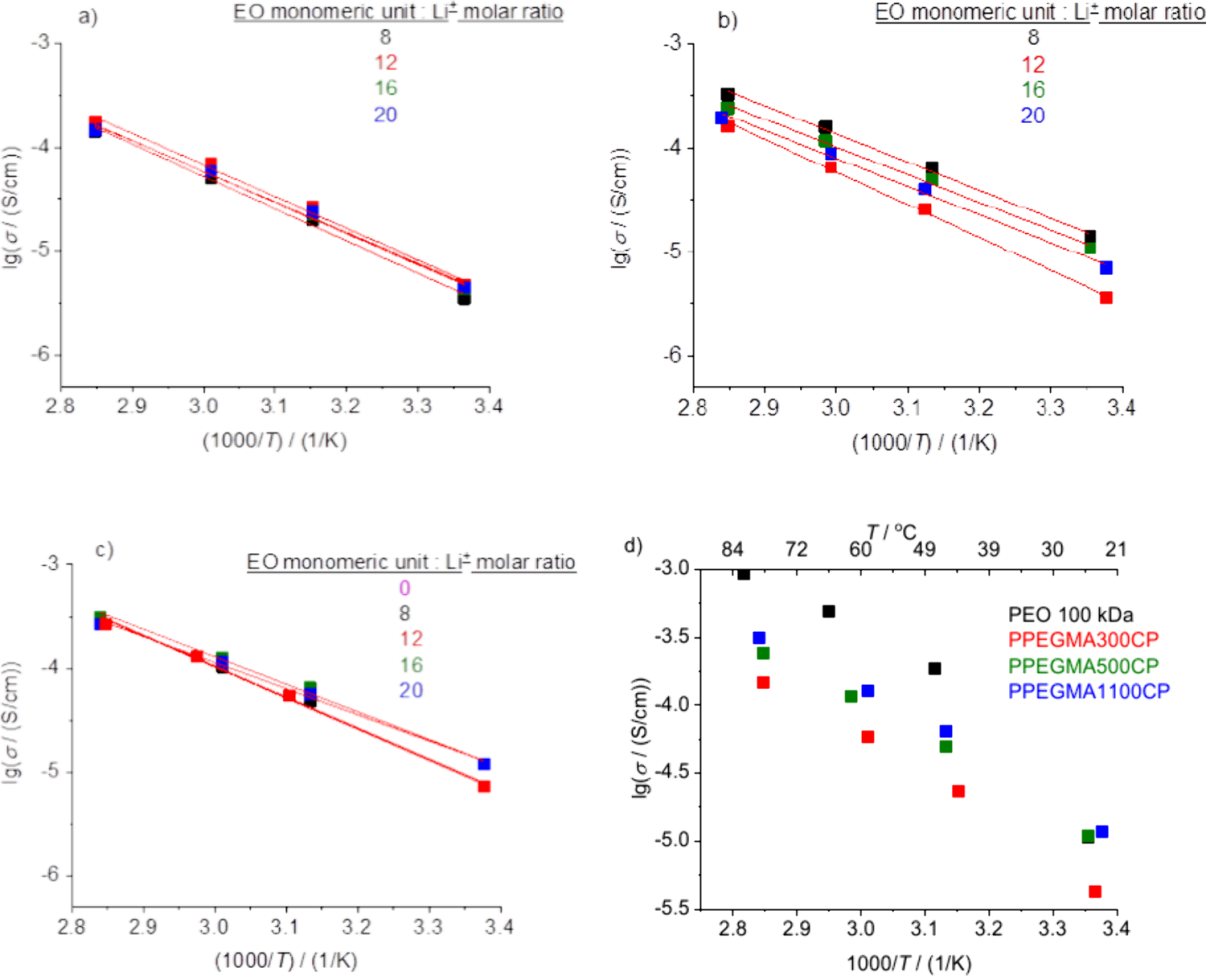
Arrhenius plots of the specific conductivity values of the PPEGMA300CP (a), PPEGMA500CP (b), and PPEGMA1100CP (c) copolymers, and the comparison of the specific conductivity values of the PEO 100 kDa sample and the PPEGMA-containing copolymers with an EO:Li^+^ molar ratio of 16:1 at different temperatures (d).

The Li-ion conductivity was determined for the dry polymer electrolyte samples by electrochemical impedance spectroscopy (EIS) measurements at four different temperatures, from ambient to 80 °C, using stainless steel blocking electrodes. The Li-salt content was varied between the EO:Li = 8 to 20 molar ratio. The obtained data were fitted with an equivalent circuit containing two Randles circuits coupled in series where the capacitances are replaced by constant phase elements (CPE) (Figure S8). While one Randles circuit with the higher resistance is assumed to describe the electrochemical behavior of the interface between the blocking-type stainless steel electrode and the salt-containing dry polymer electrolyte (*R*_int_), the one with the lower resistance was assigned to the bulk resistivity of the dry polymer electrolyte membrane (*R*_bulk_). From the latter value, the corresponding specific ionic conductivities were determined under consideration of the geometry of the given specimen. The temperature dependence of the measured specific conductivity values for the polymer electrolyte sample having different EO:Li molar ratios could be well fitted by the Arrhenius equation, as shown in Figure 5a–c. The determined ionic conductivity values fall in the 10^–6^–10^–3^ S/cm range, which is rather typical for polyether-type DSPEs. Despite the PEO side chains, the data also indicate the absence of polymer semicrystallinity at ambient temperature in the case of these comb-like copolymer mixtures with LiTFSI. This observation is in accordance with the DSC results, where crystallinity peaks were not observed upon heating and/or cooling the samples.

**Figure 6:**
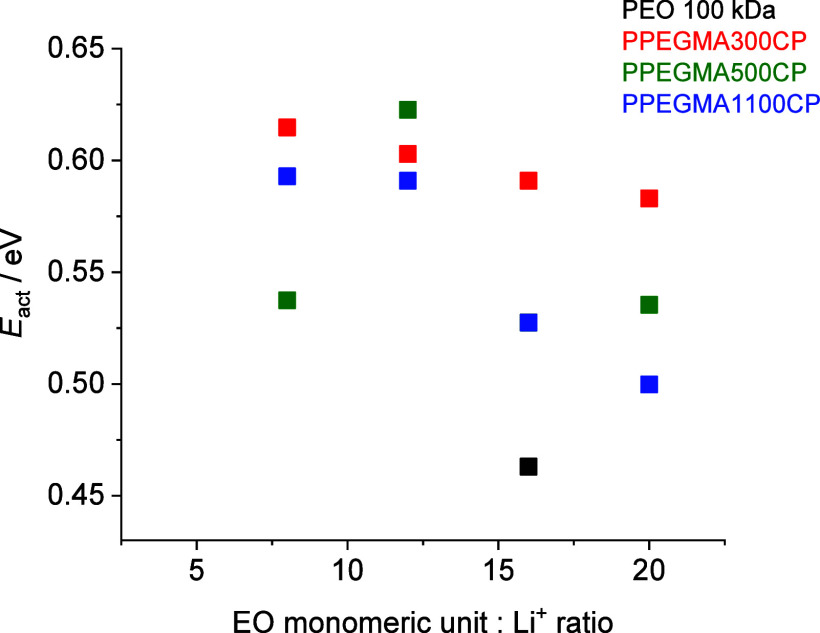
Activation energy (*E*_act_) values of the conductivity of the dry copolymer electrolytes as a function of the EO:Li molar ratio.

The Arrhenius plots, shown in Figure 5a–c, indicate that the relative amount of salt added to the polymer to form a dry solid polymer electrolyte has only a slight effect on the specific conductivity. Moreover, for comparison, the specific conductivity data were determined for a reference cell containing poly(ethylene oxide) with *M* = 100 000 g/mol (PEO 100 kDa) at a 16 EO:Li^+^ molar ratio at different temperatures (Figure 5d). As can be seen in this Figure, the PEO-based electrolyte shows higher specific conductivity at higher temperatures, i.e., above the melting temperature of the PEO 100 kDa-based electrolyte. This can be explained by the absence of the polymethacrylate backbone, which has a hampering effect on the mobility of the PEG side chains in the copolymers. However, the conductivity of PEO at ambient temperature, where its semicrystalline state blocks the motion of the polymer chains, is in the same range as for the copolymers or is even smaller than that of the PPEGMA1100CP copolymer. It should also be concluded that increasing the PEGMA side chain length results in increasing the specific conductivity. It should be considered that the PPEGMA1100CP sample has higher PMMA content (see Table 1), and this results in slightly lower specific conductivity, than that of the PPEGMA500CP and PPEGMA300CP copolymers. It should be noted that in the case of the PPEGMA copolymers, the same specific conductivity values could be reached by lower total EO content than in the linear PEO homopolymer, which is advantageous due to the decreased amount of the required Li-salt. Moreover, from the slope of the linear Arrhenius fitting, the activation energies of the Li-ion conductivity can be determined. These values are summarized in Figure 6, and as can be seen, the activation energies of the Li-ion conductivity of the copolymers are higher than that of the linear PEO homopolymer/lithium salt mixtures (0.46 eV at EO:Li^+^ = 16:1 molar ratio above the *T*_melting_ of the PEO homopolymer). The activation energy values show only a slight decreasing tendency with decreasing salt concentration, i.e. by increasing the EO monomeric unit: Li^+^ molar ratio.

**Figure 7:**
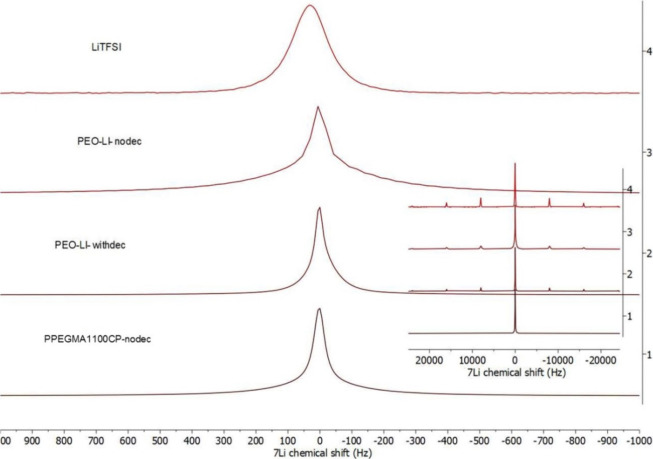
^7^Li MAS spectra at 25 °C of LiTFSI without decoupling (LiTFSI), PEO without decoupling (PEO-Li-nodec), PEO with ^19^F decoupling (PEO-Li-withdec), and PPEGMA1100CP without decoupling (PPEGMA1100CP-nodec). The full spectra are depicted in the inset (50000 Hz).

In order to clarify the negligible effect of salt concentration on the ionic conductivities, solid-state NMR studies were carried out focusing on the salt effect. Both the cation and the anion contain straightforwardly measurable NMR active nuclei, ^7^Li and ^19^F, respectively. For the NMR measurements, the PPEGMA1100CP and PEO samples containing the same salt concentration (EO:Li^+^ = 16:1 molar ratio) were chosen. The shape of the ^7^Li signal depends not only on the mobility of the Li^+^ ions but also on the couplings with hydrogen and fluorine atoms as well. In the case of the solvation of Li^+^ ions by PEO units, the fluorine coupling is negligible; however, hydrogen coupling can appear. This effect is clearly observed in the ^7^Li MAS NMR spectra. The pure LiTFSI salt has a broad signal with 137 Hz of fwhm without fluorine decoupling, as Figure 7 shows.

**Figure 8:**
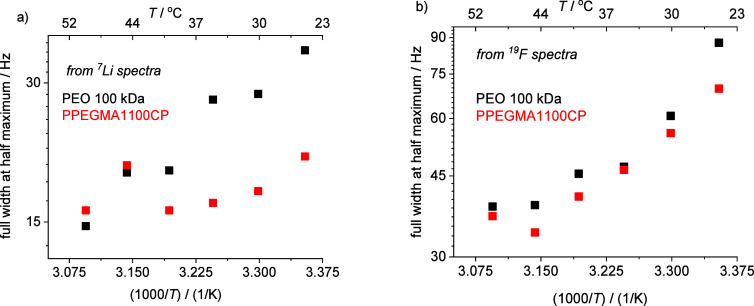
Arrhenius plots of the full width values at half-maximum of the signals belonging to the solvated ions in ^7^Li (a) and ^19^F (b) solid-state NMR spectra.

The spectrum of PEO recorded with the same conditions consists of the superposition of two overlapping signals (with 270 and 30 Hz of fwhm). The width of the broader signal can be significantly decreased by fluorine decoupling, but the narrower signal remains unchanged. However, the spectrum of PPEGMA1100CP consists of only one single narrow signal even without decoupling. The ^7^Li spectrum of PEO contains spinning sidebands with similar intensity to LiTFSI, while no sidebands are observable in the spectrum of PPEGMA1100CP. Considering these observations, it can be concluded that the narrow signal belongs to the solvated Li^+^ ions and the broad one to the not-solvated ions. This means that a significant ratio of Li^+^ ions is not solvated in the PEO homopolymer at ambient temperatures, but rather a close proximity was found between Li^+^ and fluorine atoms. Similar behavior of the ^19^F signal was also observed. Deconvolution of the spectrum shows that ca. 70% of the Li^+^ ions exist in aggregated form (ion-pair) with the anion in the PEO 100 kDa sample at room temperature. The aggregated state was also assumed previously on the basis of IR measurements.^45^ Presumably not only aggregation occurs, but the results of the solid-state NMR measurements suggest that phase separation takes place as well. The ionic conductivity is inhered to the solvated ions and their mobility was monitored by direct polarization MAS ^19^F and ^7^Li spectra as the function of temperature in the 25–50 °C range. The broader ^7^Li and ^19^F signal disappeared above 35 °C, and the full-width values at half-maximum of the narrower component decreased by increasing temperature as Figure 8 shows. The signal narrowing is monotonous with the rise of the temperature in the case of fluorine (see Figure 8b), and surprisingly, it is not monotonous in the case of Li^+^ ions (see Figure 8a). While the fluorine signal behaves similarly in the two samples, the lithium signal appears differently. At low temperatures, the ^7^Li signal is almost two times broader in PEO than in the PPEGMA1100CP environment. The origin of the different behavior can be attributed to the lower mobility of the chains in the partially crystalline PEO 100 kDa sample. At the melting point of PEO (∼45 °C), the width of the ^7^Li signal has a local maximum and the difference between the two polymeric environments disappears above this temperature. It should be noted that the spectra of PEO were found unchanged after 2 h of cooling back the sample from 52 to 25 °C. This means that the aggregation and the phase separation below 35 °C need a longer time after cooling the PEO 100 kDa sample below its melting point.

**Figure 9:**
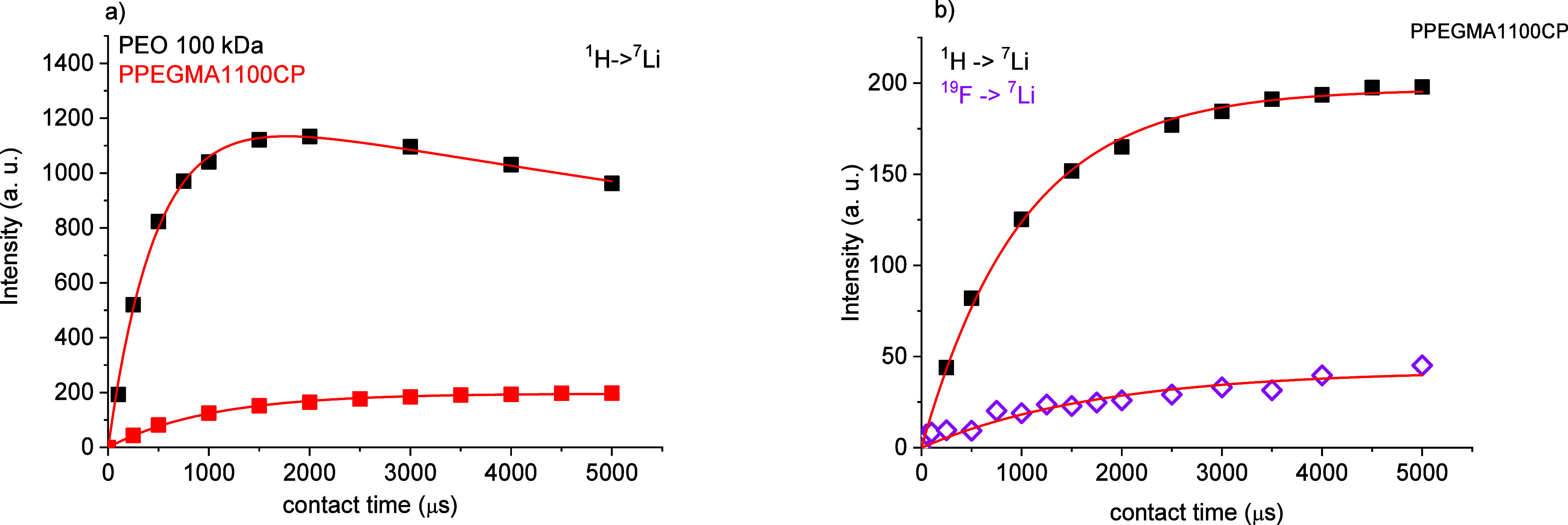
(a) ^1^H–^7^Li cross-polarization build-up curves at 25 °C and (b) the ^1^H–^7^Li and ^19^F–^7^Li cross-polarization build-up curves at 25 °C in the PPEGMA1100CP copolymer.

Cross-polarization build-up curves were also recorded to explore the heteronuclear connectivity and dynamics of Li^+^ ions at 30 °C on preheated samples to eliminate the aggregated ions (see Figure 9a,b). For fitting of the CP contact time vs intensity curves, a simplified expression was used to provide information on the mobility of Li^+^ ions and their hydrogen or fluorine environment:
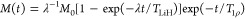
4where λ = 1 + (*T*_LiH_/*T*_1ρ_), *M*(*t*) is the magnetization at contact time *t*, *M*_0_ is the initial magnetization, *T*_LiH_ is the time coefficient of the CP (the time it takes for magnetization to be transferred from ^1^H (or ^19^F to ^7^Li), and *T*_1ρ_ is the relaxation time of the lithium in the rotating frame. This equation is valid only in a regime of fast molecular motion, but it qualitatively describes the experimental CP build-up curves and permits comparison of the fitted parameters (Table 2). The *T*_1ρ_ values show that the mobility of Li^+^ ions is much higher in PPEGMA1100CP than in the PEO sample at room temperature. The infinite value of relaxation time suggests a liquid-like behavior for the PPEGMA1100CP matrix while the cross-polarization buildup curve in PEO shows a solid-like behavior. This observation is in good agreement with the line width measurements; thus, a significant ratio of Li^+^ ions are “frozen” due to the crystalline PEO phase below the melting point of PEO, while in the PPEGMA1100CP sample, no “trapped” Li^+^ ions were detected. Comparison of cross-polarization buildup curves of ^1^H–^7^Li and ^19^F–^7^Li in the PPEGMA1100CP sample offers the possibility to investigate the solvation of Li^+^ ions. The curves clearly show that Li^+^ ions are complexed by the EO units in the side chains in the PPEGMA1100CP sample. While the *T*_1ρ_ relaxation parameters are infinite in both cases, the *T*_LiH_ coefficient is definitively smaller than the *T*_LiF_ coefficient; thus, the F–Li connections are much weaker than the H–Li connection but not negligible. This indicates that the fluorine atoms are present most probably not in the first solvate shell.

**Table 2:** Fitted Parameters of the Cross-Polarization Build-Up Curves Shown in Figure 9

**polymer**	PEO	PPEGMA1100CP	PPEGMA1100CP
*M*_0_/a.u.	1252	197	42
X nucleus	^1^H	^1^H	^19^F
*T*_LiX_/μS	504	1007	1782
*T*_1ρ_/μS	17609	7.7 × 10^20^	1.2 × 10^21^

**Figure 10:**
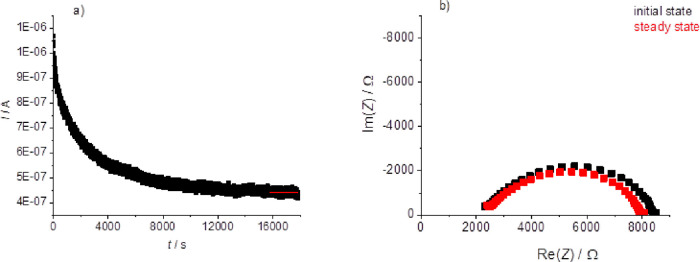
Current–time (a) and the EIS (b) curves in the initial state and in the steady state of the PPEGMA1100CP based electrolyte with a 16 EO/Li^+^ molar ratio during the transference number determination measurement performed at 70 °C.

**Figure 11:**
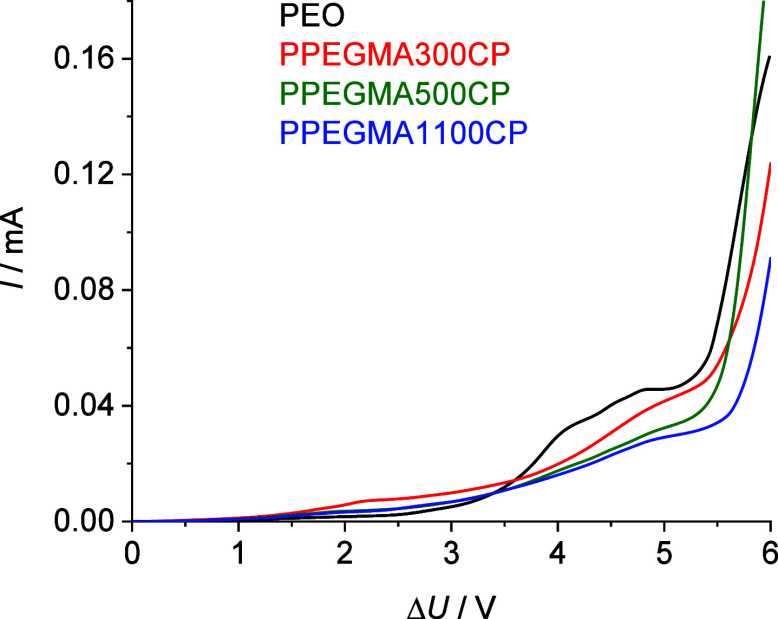
Linear sweep polarization curves for the electrochemical stability of the symmetrical cells containing the polymer electrolytes.

In relation to the electrochemical behavior, the Li-ion transference numbers were determined by the method reported by Evans et al.^39^ (Figure 10a,b). In the case of the copolymer with 23 EO unit side chains (i.e., PPEGMA1100CP) this value was 0.19 at 70 °C, similar to the PEO homopolymer, which is slightly higher (0.21) in accordance with the value reported earlier.^21^ Electrochemical stability measurements were also performed for all the investigated polymer electrolytes using a 16:1= EO:Li molar ratio. In these linear sweep voltammetry (LSV) measurements, the potential difference between the two blocking electrodes was increased from 0 to 6 V while the current was recorded. As can be seen in Figure 11, all of these copolymers proved to be more stable against electrochemical degradation than the PEO 100 kDa homopolymer since a considerable current increment occurs at higher voltage. The extrapolated onset value of the decomposition for the PEO 100 kDa, PPEGMA300CP, PPEGMA500CP, and PPEGMA1100CP were found to be 5.16 V, 5.39 V, 5.45 and 5.48 V, respectively. This suggests that the PPEGMA copolymer electrolyte samples exhibit 200–300 mV wider electrochemical stability window than the PEO homopolymer.

## Conclusions

Unique copolymers of poly(poly(ethylene glycol) methacrylate) with methyl methacrylate were successfully synthesized by one-pot ATRP. It was found that the obtained structure provides multiple benefits for the DSPE electrolyte. The polymethacrylate backbone assures good mechanical properties even at elevated temperatures for the copolymers and suppresses the crystallization of the polyether-like poly(ethylene glycol) side chains. Furthermore, the wetting property analyses demonstrated that the obtained amphiphilic copolymers, based on their chemical composition, possess mixed hydrophilic (PPEGMA) and hydrophobic (PMMA) wetting properties. This latter can be fine-tuned by alteration of the chemical composition of the copolymers. This feature may be exploited later by the bulk-type composite electrode formulation, where functional particles with different wetting properties must be dispersed in the continuous polymer-type Li-ion conductive electrolyte matrix. Mechanical characterization of the copolymers showed that they behave as viscoelastic polymer melts in the investigated temperature range and do not exhibit semisolid-type behavior due to the suppressed crystallinity, which favors the segmented dynamic determined Li-ion conductivity. The temperature-dependent Li-ion conductivity analyses showed that the conductivity falls into the 10^–6^–10^–3^ S/cm range, which is typical for polyether-type DSPEs but with much lower mass fractions of EO monomers in the copolymers providing the same ionic conductivity values as the PEO homopolymer. From a large-scale practical point of view, this is in direct relationship with the reduced Li-salt usage if PPEGMA-type matrices are used instead of PEO. Moreover, linear sweep voltammetry (LSV) polarization measurements showed that the PPEGMA-MMA copolymer electrolytes can exhibit a 200–300 mV broader electrochemical stability window than PEO, which can be attributed to the chemical structure of the copolymers compared to the homopolymer. Nevertheless, the determined Li-ion transference number for the copolymers is very similar to that of the PEO homopolymer, and the LiTFSI concentration has a slight effect on the ionic conductivities. This was further analyzed by ^7^Li MAS direct polarization NMR studies, which demonstrated that in the pure PEO samples, a certain portion of the Li-ions is trapped (“frozen”) into the crystalline PEO below the melting point, contrary to the copolymers of PPEGMA brushes with MMA. Furthermore, the ^1^H–^7^Li NMR cross-polarization build-up curves corroborate this finding. At low temperatures, the mobility of the Li^+^ ions is slower in PEO homopolymer, because the segment motions of the PEO chains are clogged by the crystallization. In contrast, no such effect is observed for the PPEGMA1100 copolymer. No evidence of ion pair formation was seen in the NMR spectra of this sample even at lower temperatures. These findings enable us to consider the copolymers of the PPEGMA brushes with MMA as a potential novel class of efficient Li-ion conducting matrices for future generations of Li-ion batteries.

## Supporting Information Available

Macroinitiator synthesis description, ^1^H NMR spectra and gel permeation (GPC) chromatograms of the synthesized polymers, and drawing of the equivalent circuit (PDF)
