# Supplementary material for: Nonionic Amphiphilic Copolymers of Poly(poly(ethylene Glycol) Methacrylate) Brushes with Methyl Methacrylate Prepared by Atom Transfer Radical Polymerization as Dry Solid Polymer Electrolytes for Next Generation Li-ion Battery Applications
Source: ACS Appl Energy Mater. 2024 Dec 9;7(24):12036–47. doi: 10.1021/acsaem.4c02519 (PMC11673846; doi:10.1021/acsaem.4c02519)
Supplement: Supplementary file 1 — ae4c02519_si_001.pdf [file ae4c02519_si_001.pdf]

SUPPORTING INFORMATION

Non-ionic amphiphilic copolymers of poly(poly(ethylene glycol) methacrylate) brushes with methyl methacrylate prepared by ATRP as dry solid polymer electrolytes for next generation Li-ion battery applications alma

Ákos Szabó[a], Denis Ershov[b], Ágnes Ábrahám[c,d], Éva Kiss[d], Györgyi Szarka[a], Ilona Felhősi[e], Benjámin Gyarmati[f], Attila Domján[g,h], Béla Iván[a,*], Robert Kun[b,i,*]

[a] *Polymer Chemistry and Physics Research Group, Institute of Materials and Environmental Chemistry, HUN-REN Research Centre for Natural Sciences, H-1117 Budapest, Magyar tudósok krt. 2., Budapest, Hungary*

[b] *Department of Chemical and Environmental Process Engineering, Faculty of Chemical Technology and Biotechnology, Budapest University of Technology and Economics, Műegyetem rkp. 3, H-1111 Budapest, Hungary*

[c] *MTA-TTK Lendület "Momentum" Peptide-Based Vaccines Research Group, Institute of Materials and Environmental Chemistry, HUN-REN Research Centre for Natural Sciences, H-1117 Budapest, Magyar tudósok krt. 2.*

[d] *Laboratory of Interfaces and Nanostructures, Institute of Chemistry, Eötvös Loránd University, 112, PO Box 32, H-1518 Budapest, Hungary*

[e] *Functional Interfaces Research Group, Institute of Materials and Environmental Chemistry, HUN-REN Research Centre for Natural Sciences, H-1117 Budapest, Magyar tudósok krt. 2., Budapest, Hungary*

[f] *Soft Matters Group, Department of Physical Chemistry and Materials Science, Faculty of Chemical Technology and Biotechnology, Budapest University of Technology and Economics, H-1111 Budapest, Műegyetem rkp. 3, Hungary*

[g] *Current address: AGES - Austrian Agency for Health and Food Safety, Wieningerstr. 8, 4020 Linz, Austria*

[h] *Centre for Structural Science, HUN-REN Research Centre for Natural Sciences, Magyar tudósok krt. 2, 1117 Budapest, Hungary*

[i] *Solid-State Energy Storage Research Group, Institute of Materials and Environmental Chemistry, HUN-REN Research Centre for Natural Sciences, H-1117 Budapest, Magyar tudósok krt. 2., Budapest, Hungary*

*Corresponding authors: Prof. Béla Iván, Dr. Robert Kun

E-mail adresses: ivan.bela@ttk.hu (B.I.) kun.robert@ttk.hu (R.K.)

Synthesis of the bifunctional telechelic PEG macroinitiator

Bifunctional PEG-bis(2-bromoisobutyrate) initiator was synthesized by dissolving 4.4 ml of PEG (400 g/mol) in distilled tetrahydrofuran containing 6.1049 g N,N-dimethylaminopyridine, and then adding solution of 6.18 ml 2-bromoisobutyryl bromide in 10 ml tetrahydrofuran dropwise in the reaction mixture while it was cooled with water bath. Subsequently, 25 ml tetrahydrofuran was added and the mixture was allowed to be stirred overnight. On the next day, 150 ml dichloromethane was added, the solution was filtered by filter paper, and was extracted with concentrated $NaHCO_3$ solution three times followed by drying on $MgSO_4$. Then it was filtered again by filter paper, and the solvent was evaporated by rotavap. The product was dried in vacuum at room temperature.

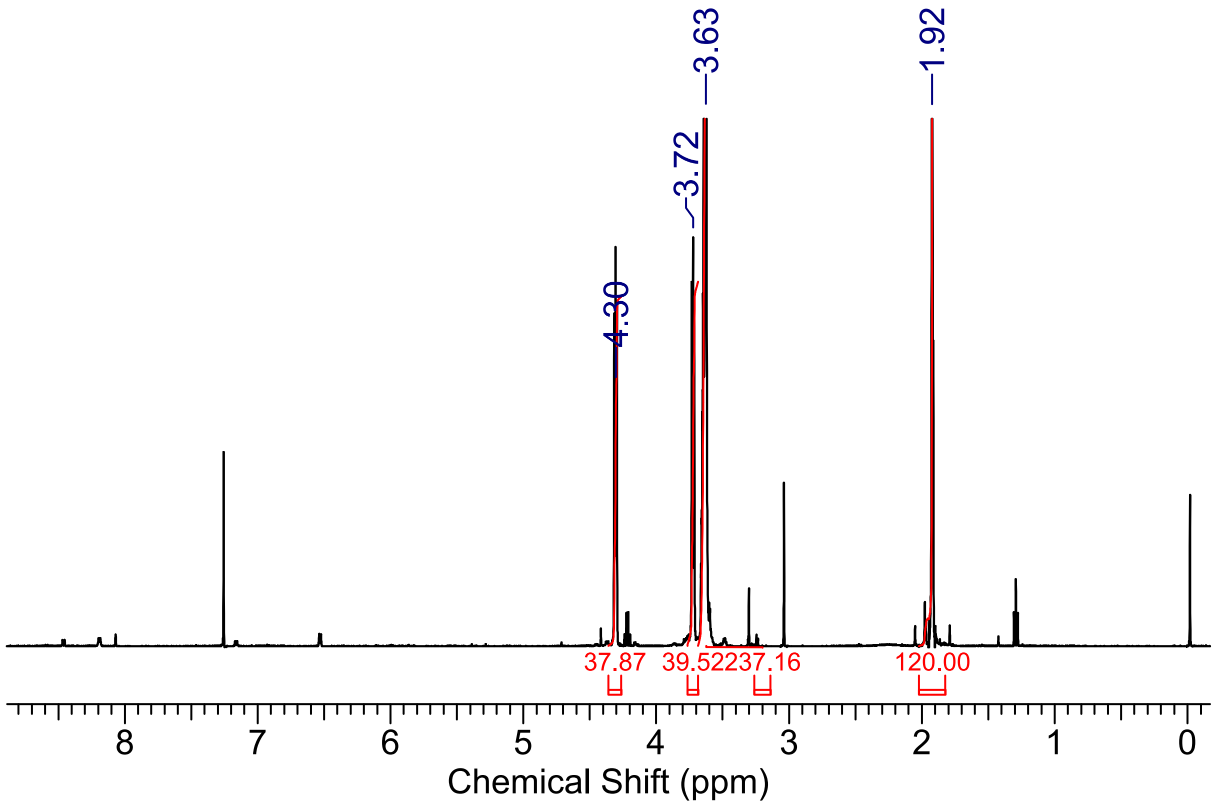

Figure S1. The ^{1}H NMR spectrum of the bifunctional telechelic PEG macroinitiator. The signal at 4.30 ppm belongs to the –C**H₂**- groups of the PEG segment adjacent to the ester groups, the signals between 3.5 and 3.8 ppm to the other –C**H₂** groups of the PEG segment while the signal at 1.92 ppm to the –C**H₃**- groups of the bromoisobutyrate moieties.

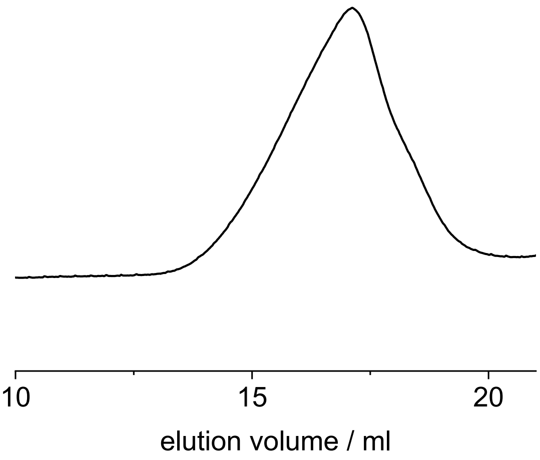

Figure S2. The GPC chromatogram of the PPEGMA300CP copolymer.

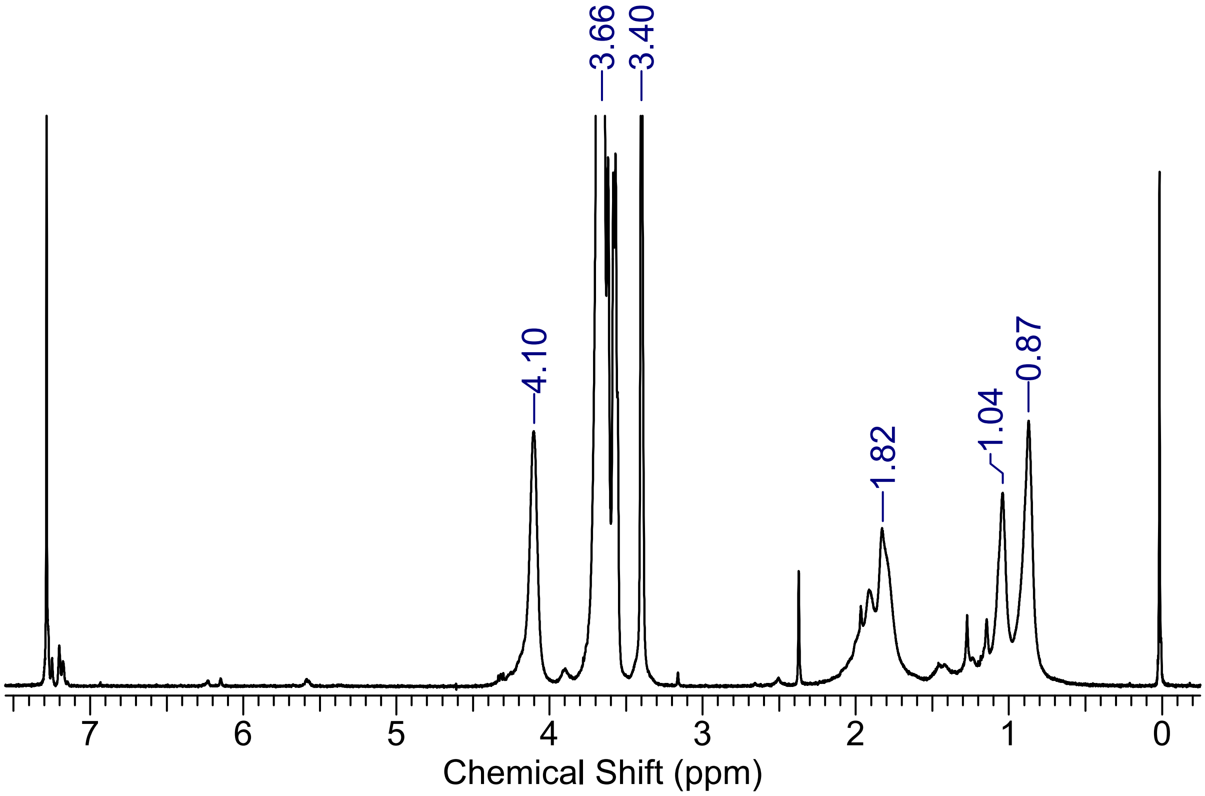

Figure S3. The ^{1}H NMR spectrum of the PPEGMA300CP copolymer.

The signal at 4.10 ppm belongs to the -C**H**$_2$- group adjacent to the ester group in the side chain of the PPEGMA monomeric units, the signals between 3.5 and 3.8 ppm to the other –C**H**$_2$ groups of these side chains, the signal at 3.40 ppm to the ω-C**H**$_3$ group of these side chains while the signals between 1.6 and 2.1 ppm to the –C**H**$_2$-

groups of the copolymer backbone, and the signals between 0.6 and 1.6 ppm to the –CH_3 groups of the copolymer backbone.

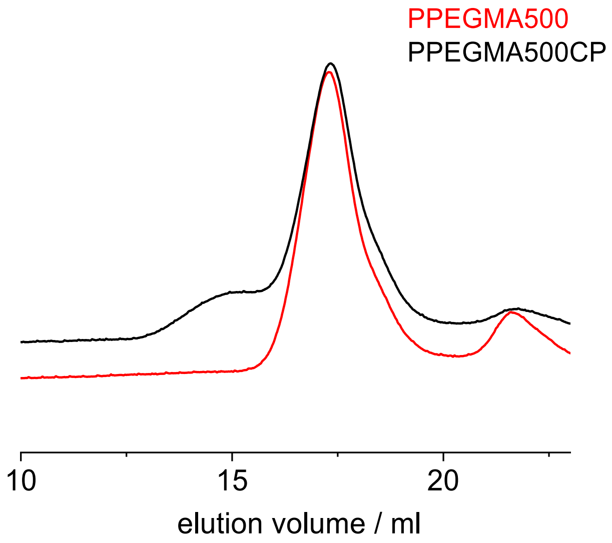

Figure S4. The GPC chromatograms of the PPEGMA500CP copolymer and its PPEGMA500 segment.

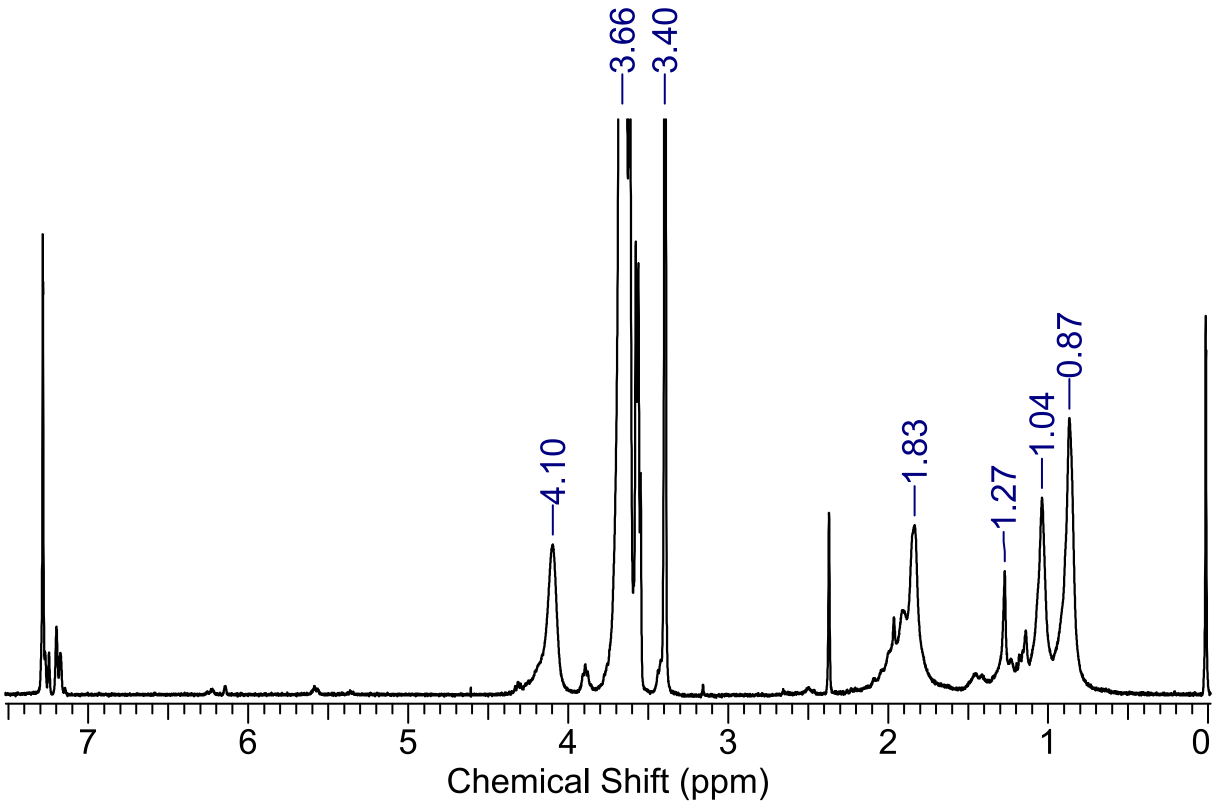

Figure S5. The ^{1}H NMR spectrum of the PPEGMA500CP copolymer.

The signal at 4.10 ppm belongs to the -C**H$_2$**- group adjacent to the ester group in the side chain of the PPEGMA monomeric units, the signals between 3.5 and 3.8 ppm to the other –C**H$_2$** groups of these side chains, the signal at 3.40 ppm to the ω-C**H$_3$** group of these side chains while the signals between 1.6 and 2.1 ppm to the –C**H$_2$**- groups of the copolymer backbone, and the signals between 0.6 and 1.6 ppm to the –C**H$_3$** groups of the copolymer backbone.

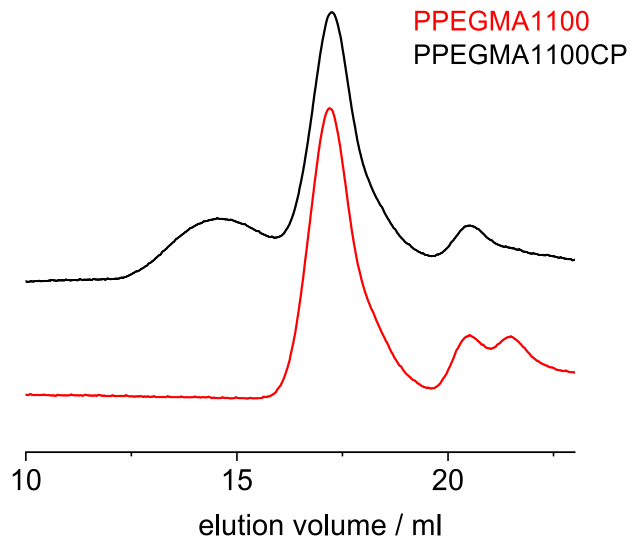

Figure S6. The GPC chromatograms of the PPEGMA1100CP copolymer and its PPEGMA1100 segment.

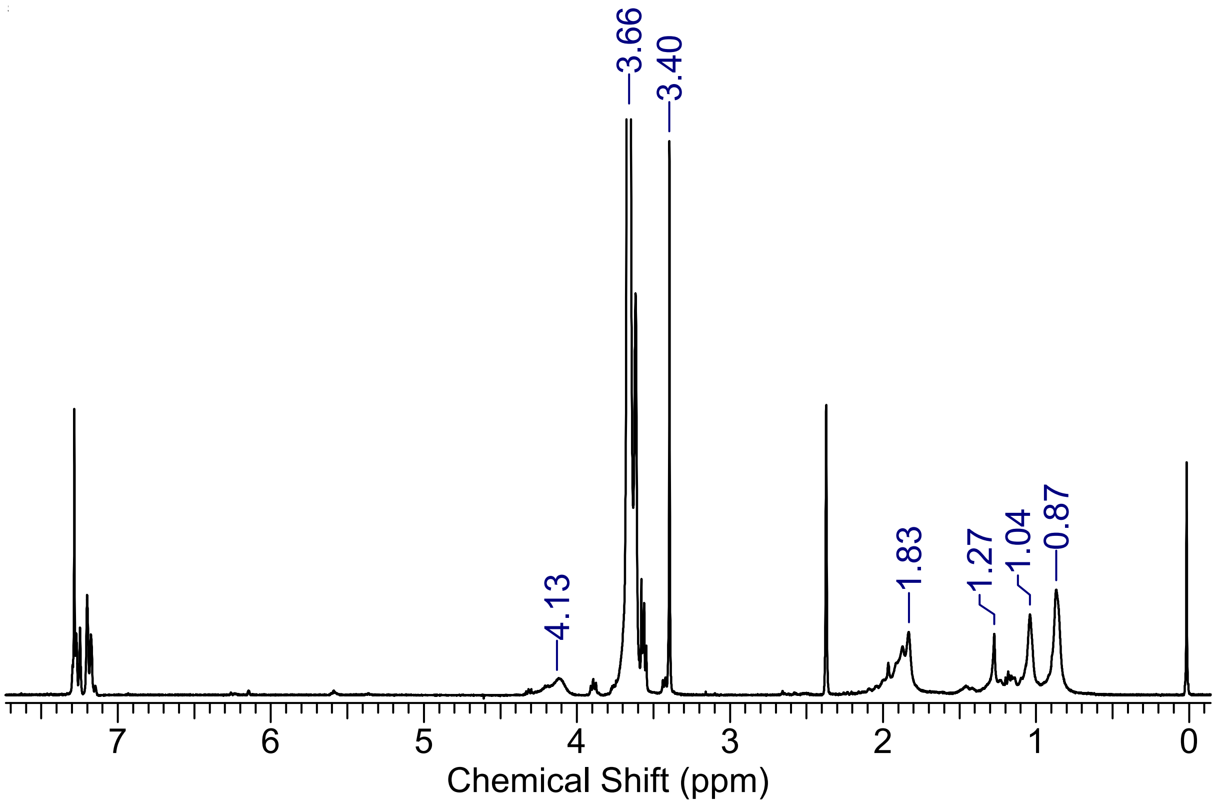

Figure S7. The ^{1}H NMR spectrum of the PPEGMA1100CP copolymer.

The signal at 4.10 ppm belongs to the -CH_2- group adjacent to the ester group in the side chain of the PPEGMA monomeric units, the signals between 3.5 and 3.8 ppm to the other –CH_2 groups of these side chains, the signal at 3.40 ppm to the ω-CH_3 group of these side chains while the signals between 1.6 and 2.1 ppm to the –CH_2- groups of the copolymer backbone, and the signals between 0.6 and 1.6 ppm to the –CH_3 groups of the copolymer backbone.

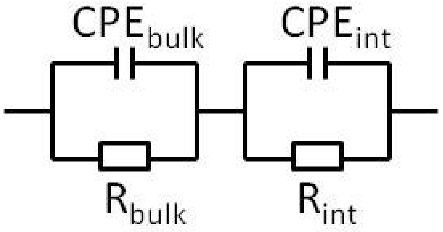

Figure S8. Equivalent circuit model used to fit the electrochemical impedance (EIS) spectra in order to determine the conductivity of the electrolyte. R_{bulk}: resistance of the bulk phase of polymer electrolyte, CPE_{bulk}: non-ideal geometric capacitance, R_{int}: interface resistance, CPE_{int}: non-ideal capacitance of the interface
